# Poxvirus Cell Entry: How Many Proteins Does it Take?

**DOI:** 10.3390/v4050688

**Published:** 2012-04-27

**Authors:** Bernard Moss

**Affiliations:** Laboratory of Viral Diseases, National Institute of Allergy and Infectious Diseases, National Institutes of Health, Bethesda, MD 20892, USA; Email: bmoss@nih.gov; Tel.: +1-301-496-9869; Fax: +1-301-480-1535

**Keywords:** vaccinia virus entry, viral membrane fusion, endocytosis, macropinocytosis, transmembrane proteins

## Abstract

For many viruses, one or two proteins enable cell binding, membrane fusion and entry. The large number of proteins employed by poxviruses is unprecedented and may be related to their ability to infect a wide range of cells. There are two main infectious forms of vaccinia virus, the prototype poxvirus: the mature virion (MV), which has a single membrane, and the extracellular enveloped virion (EV), which has an additional outer membrane that is disrupted prior to fusion. Four viral proteins associated with the MV membrane facilitate attachment by binding to glycosaminoglycans or laminin on the cell surface, whereas EV attachment proteins have not yet been identified. Entry can occur at the plasma membrane or in acidified endosomes following macropinocytosis and involves actin dynamics and cell signaling. Regardless of the pathway or whether the MV or EV mediates infection, fusion is dependent on 11 to 12 non-glycosylated, transmembrane proteins ranging in size from 4- to 43-kDa that are associated in a complex. These proteins are conserved in poxviruses making it likely that a common entry mechanism exists. Biochemical studies support a two-step process in which lipid mixing of viral and cellular membranes is followed by pore expansion and core penetration.

## 1. Introduction

Poxviruses comprise a family of genetically related, large, enveloped, DNA viruses that replicate exclusively within the cytoplasm of vertebrate or invertebrate cells [1]. Homologs of approximately 100 of the 200 or more genes are present in all Chordopoxviruses and 50 are recognizable in both Chordopoxviruses and Entomopoxviruses [2]. The products of the highly conserved genes are involved in cell entry, gene expression, DNA replication, intramolecular disulfide bond formation and virion assembly; products of the less conserved genes participate in specific host interactions. The most intensively studied poxviruses belong to the Orthopoxvirus genus, including variola virus (causative agent of smallpox, eradicated from nature), vaccinia virus (VACV; the modern smallpox vaccine, now endemic in Brazil), cowpox virus (the original smallpox vaccine, indigenous in Europe, occasionally infects humans) and monkeypox virus (indigenous in Africa, causes a smallpox-like disease of humans). Studies of VACV have provided most of what we know about poxvirus entry [3].

For historical reasons, VACV genes and open reading frames are commonly identified with a capital letter (representing a HindIII restriction endonuclease genome fragment), an arabic number (representing the position within the HindIII fragment) and L or R (indicating transcription to the left or right, respectively). Proteins have the corresponding designation except that L or R is omitted. For example L1 is the protein encoded by L1R. The www.poxvirus.org is a useful website for correlating these common names, which are listed for the Copenhagen strain of VACV, with GenBank annotations for other VACV strains such as Western Reserve (WR).

## 2. Poxvirus Replication Cycle

**Figure 1:**
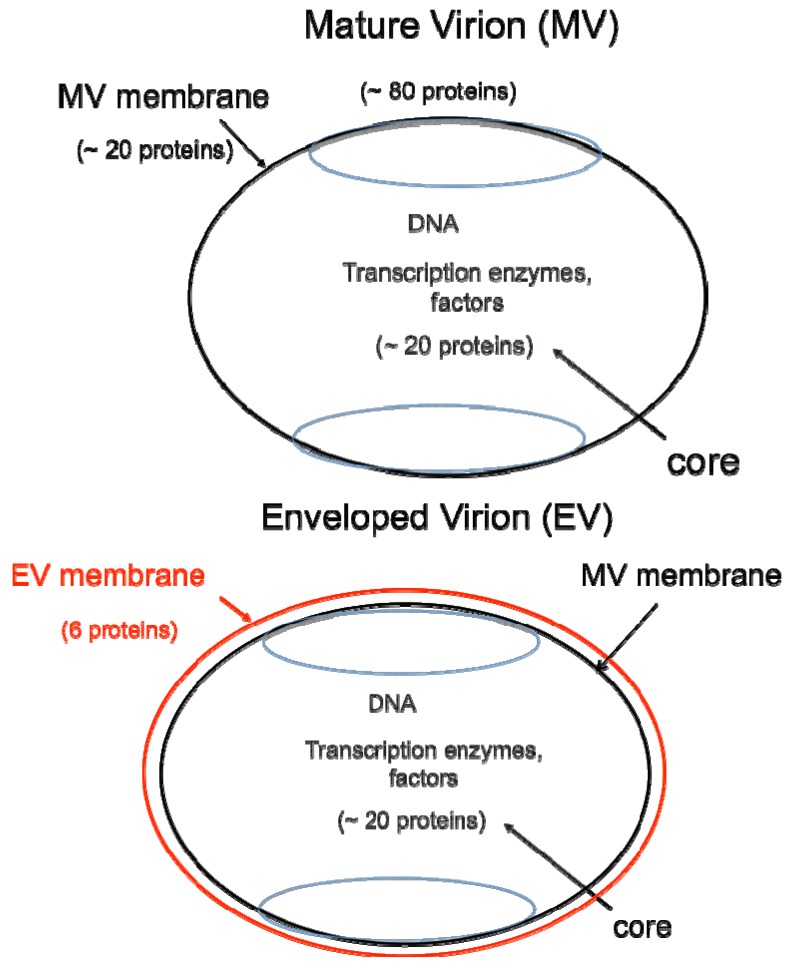
Two major forms of infectious virions. The mature virion (MV) contains more than 80 proteins and consists of a nucleoprotein core surrounded by a lipid membrane (**black**) with about 20 proteins. Approximately 20 proteins within the core are devoted to synthesis and modification of mRNA. The enveloped virion (EV) consists essentially of a MV with an additional membrane (**red**) containing about 6 proteins distinct from those in the MV membrane.

DNA viruses typically replicate and express their genomes in the nucleus making extensive use of cellular proteins. In contrast, poxviruses rely heavily on virus-encoded proteins enabling them to replicate in the cytoplasm [1]. The infectious VACV membrane-bound particle contains a core, within which reside the linear, double-stranded DNA genome and virus-encoded enzymes and factors that allow transcription of the early set of genes. When the core enters the cytoplasm, early mRNA and protein synthesis occur, followed by DNA replication [4a]. The replicated DNA provides a template for the synthesis of intermediate and late classes of mRNA. The most recent analysis of VACV WR suggests that there are 118 early, 53 intermediate and 38 late genes [4b]. Following late gene expression, virus assembly begins. The initial infectious form, called a mature virions (MV) [5], has a single external membrane [6,7,8] (Figure 1). Some MVs are wrapped in a modified trans-Golgi or endosomal membrane to become triple-membrane particles called wrapped virions (WVs), whereas other MVs remain free or in inclusions within the cytoplasm until liberated by cell lysis [9,10,11]. The WVs are transported on microtubules to the periphery of the cell where the outer membrane fuses with the plasma membrane to release an extracellular enveloped virion (EV), consisting essentially of a MV with one additional membrane [12,13] (Figure 1). At least 20 proteins are associated with the MV membrane and 6 others with the outer EV membrane. In older papers and some recent ones, MVs may be referred to as INVs or IMVs; WVs as IEVs; and EVs as EEVs and CEVs.

MVs are very stable and are thought to mediate transmission between host animals, whereas EVs have a fragile outer membrane and are specialized for exiting the intact cell and spreading within the host. EVs can remain associated with the tips of actin-containing protrusions at the cell surface or released into the surrounding fluid but the former are predominant in most VACV strains and are largely responsible for cell-to-cell spread [14,15,16,17,18]. However, a few VACV strains, such as IHD-J, release relatively large numbers of EVs [19]. Enhanced EV release may be a tissue culture adaptation of some VACV strains. The difference in the amounts of EVs released by WR and IHD-J is largely due to a single amino acid change in the A34 EV membrane protein [20]. Mutations of other EV proteins can also increase EV release [21]. Partly for technical reasons, the majority of entry studies have been carried out with MVs, usually of the WR VACV strain. A high percentage of EVs that are released from cells have a partly disrupted outer membrane, making experiments with these particles difficult to interpret. There have been virtually no investigations of entry mediated by cell-associated EVs, despite their biological importance. 

## 3. Entry Pathways

Entry of the core or nucleoprotein of enveloped viruses requires fusion of the viral membrane with either the plasma or endosomal membrane [22]. The latter route is thought to provide an advantage in providing passage through the dense cell cortex. For VACV, it is necessary to consider the existence of two infectious forms of virus, the single-membrane MV and the double-membrane EV. On theoretical grounds alone it seemed unlikely that the outer EV membrane would fuse since that would leave a membrane bound MV-like particle in the cytoplasm. Indeed, some electron microscopic images show fusion of the single MV membrane with the plasma membrane of the cell [23,24,25]. Moreover, electron microscopic images show MVs fusing with plasma membrane after dissolution of the EV membrane, apparently by interaction with glycosaminoglycans at the cell surface [26]. 

**Figure 2:**
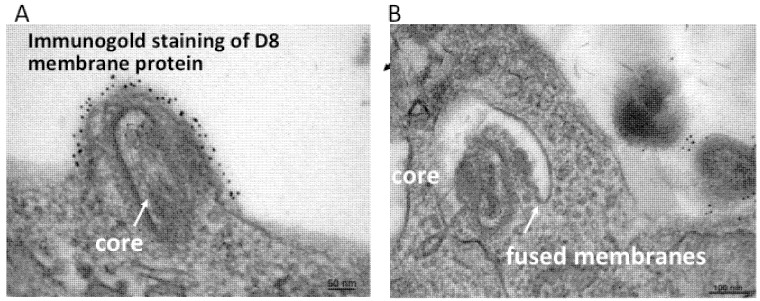
Transmission electron micrographs showing a VACV MV fusing with the plasma membrane (**A**) and endosomal membrane (**B**). Prior to cryosectioning, the infected cells were stained with a monoclonal antibody to the MV membrane protein D8 followed by protein A conjugated to gold spheres. Modified from reference [25].

**Figure 3:**
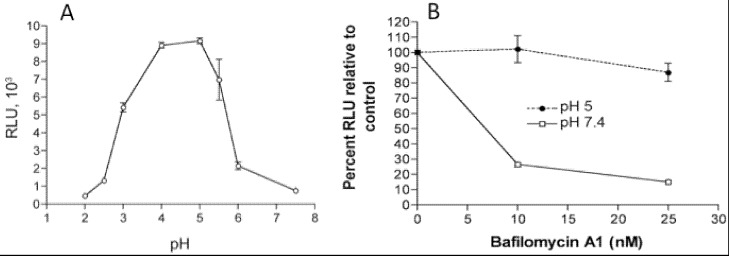
Effects of low pH and inhibitors of endosomal acidification on VACV MV entry. (**A**) Following adsorption of a recombinant VACV MV that expresses firefly luciferase at 4 °C, cells were incubated with buffers at the indicated pH for 3 min at 37 °C. The cells were then incubated with regular medium for 1 h at 37 °C, lysed and luciferase activity measured; (**B**) Cells were pretreated with indicated concentrations of bafilomycin A1 for 50 min at 37 °C and then infected with MVs, briefly exposed to pH 5 or 7.4 buffer, and luciferase activity measured as in panel A. Note that incubation at pH 5 allows fusion at the plasma membrane and bypasses the effect of the inhibitor. Modified from reference [25].

In addition to the above images of VACV fusing with the plasma membrane, other images show MVs within endosomes [27,28]. Further microscopic (Figure 2) and biochemical studies indicate that VACV MVs can enter cells via neutral pH plasma membrane or low pH endocytic pathways [25]. The strong enhancement of entry following brief low pH (<6) treatment of cell-bound virions (mimicking the low pH of late endosomes) and inhibition of entry by preventing acidification of endosomes (Figure 3) suggests that the endocytic route is dominant for the WR strain of VACV [25]. 

Low pH as well as brief proteinase treatment can stably activate MVs prior to their adsorption to cells suggesting a triggering or unmasking effect, but these procedures do not alleviate sensitivity to inhibitors of endosomal acidification [29]. Entry of some other VACV strains, such as IHD-J, are not accelerated by low pH or inhibited by lysomotropic agents indicating entry through the plasma membrane or a neutral pH endocytic route [30]. Adaptation of entry pathways could have occurred during extensive cell culture passaging of VACV strains. Several recently isolated orthopoxviruses have been shown to be more similar to WR than IHD-J with regard to entry pathway, emphasizing the importance of the endocytic route (Z. Bengali, P.S. Satheshkumar and B. Moss, unpublished). There have been few studies of members of other poxvirus genera. Myxoma virus entry is not enhanced by low pH [31]. 

Recent evidence suggests that the VACV A25 and A26 proteins serve as fusion suppressors for MVs and determine strain-specific virus entry pathways [32]. Thus, MVs containing functional A25 and A26 proteins do not fuse readily with the plasma membrane and enter through the endocytic pathway, whereas those missing these proteins enter through the plasma membrane. Further discussion of these proteins and mechanistic aspects of the model will be presented below. The route of infection also depends on the cell type [30,33]. VACV can even enter insect cells, though replication is abortive [34,35,36]. Entry into Drosophila S2 cells is exclusively through the endocytic route [36].

Engulfment of the large VACV MV particles occurs by clathrin- and caveolin-independent macropinocytosis or fluid phase endocytosis and is dependent on actin dynamics and cell signaling [37,38,39]. The requirement for cell signaling may have led to earlier proposals that chemokine receptors serve as poxvirus entry portals [40,41,42]. Two cellular proteins, VPEF and CD98, associated with lipid rafts participate in the fluid phase uptake of MVs [37,43]. EVs may enter through the plasma membrane or following endocytosis [26,44,45]. 

The infectivity of intact and detergent extracted MVs can be enhanced by incubation with liposomes containing phosphatidylserine [46,47]. This finding was recently confirmed and extended by Mercer and Helenius [37], who also showed that annexin 5 inhibits VACV entry. They proposed that the role of phosphatidylserine in MV endocytosis is similar to the role of this lipid in uptake of apoptotic bodies. The infectivity of lipid-extracted MVs could also be enhanced by the non-biologically relevant D-stereoisomer of phosphatidylserine as well as by other phospholipids that are not known to signal uptake of apoptotic debris [48]. Therefore, the putative lipid receptor would have low specificity suggesting a role for bridging molecules. A soluble protein called Gas6 can bridge phosphatidylserine and the TAM receptor tyrosine kinase Axl on certain cells [49]. However, Gas6 had little or no effect on MV entry, although EV entry was enhanced [49]. Paradoxically, annexin 5 was reported not to inhibit EV entry, suggesting that phosphatidylserine is not involved [45]. Further studies are needed to understand the role of lipids in VACV entry.

## 4. Attachment

Proteinase treatment of cells can prevent binding of MVs suggesting a role for cell surface proteins [50,51]. Four viral proteins can mediate MV attachment. D8 binds chondroitin sulfate [52] and A27 and H3 proteins bind heparan sulfate [53,54,55,56] indicating the importance of glycosaminoglycans (Table 1). Heparin appears to have a greater inhibitory effect on VACV strains that preferentially enter by a neutral pH mechanism [30]. The A26 protein binds laminin [57]. Of the four attachment proteins, only H3 is highly conserved among poxviruses. D8 and H3 have transmembrane domains; the A26 and A27 proteins interact with each other and the latter is anchored by the A17 transmembrane protein, which is an important structural element of the MV membrane [58,59,60]. The attachment proteins are multifunctional and are not individually essential, though deletion of A27 and H3 severely reduce VACV infectivity [55,61,62]. H3 also participates in MV assembly and A27 is required for formation of the WV. In addition, the A26 protein mediates the incorporation of MVs into so-called A-Type cytoplasmic inclusion bodies of some orthopoxviruses including cowpox virus and ectromelia virus [63]. The A25 protein of VACV, recently described as a fusion suppressor [32], is a truncated form of the A-type inclusion protein [64,65]. A soluble, truncated, recombinant form of the L1 protein, described below, can attach to cells lacking glycosaminoglycans and prevent VACV entry suggesting that it is a receptor binding protein [66]. However, the putative cellular protein interacting with L1 remains to be determined. The binding of EVs was not diminished by proteinase treatment of cells and the attachment molecules have not been identified [50].

**Table 1:** VACV MV attachment and entry proteins.

Protein	kDa	TM ^a^	Expr ^b^	Cons ^c^	Properties
Attachment					
A26	58	-	L	-	Binds laminin; assoc. with A27
A27	13	-	I	-	Binds heparan; assoc with A17; N ^d^
D8	35	N	I	-	Binds chondroitin; N
H3	38	C	I	P	Binds heparan; N
Entry					
A16	43	C	I	P	EFC ^e^; paralog G9, J5; binds G9; C-C ^f^
A21	14	N	L	P	EFC; C-C
A28	16	N	L	P	EFC; N; binds H2; C-C
F9	24	C	L	P	EFC associated; C-C
G3	13	N	L	P	EFC; binds L5
G9	39	C	L	P	EFC; paralog A16, J5; binds A16; C-C
H2	22	N	L	P	EFC; binds A28; C-C
I2	8	C	L	C	EFC?
J5	15	C	L	P	EFC; paralog A16, G9; C-C
L1	27	C	L	P	EFC associated; N; C-C; Myr ^g^
L5	15	C	L	P	EFC; binds G3; C-C
O3	4	N	I	C	EFC

^a^ TM, N- or C-terminal transmembrane domain; ^b^ Expr, expressed at I (intermediate) or L (late) times of replication; ^c^ Cons, conserved in all poxviruses (P) or all chordopoxviruses (C); ^d ^N, target of neutralizing antibody; ^e^ EFC, component of entry-fusion complex; ^f^ C-C, intramolecular disulfide bond(s); ^g^ Myr, myristoylated.

## 5. Identification of Viral Proteins that Mediate Core Entry

Although many enveloped viruses encode one or two proteins that are sufficient to mediate attachment and entry, VACV is exceptional. Thus far, 11 to 12 proteins have been implicated in a post-attachment entry step (Table 1). These proteins are conserved in all poxviruses with the possible exception of O3; although the small size of the latter protein makes it difficult to detect statistically significant homology, all chordopoxviruses encode a protein with similar features at the same genome location as O3 [67]. Entry proteins were originally discovered by identifying genes predicted to encode transmembrane proteins that are conserved in all poxviruses, constructing inducible mutants, and determining their phenotypes [68,69,70]. MVs that formed in the absence of inducer, and therefore lacking the specific target protein, could bind to cells but had low infectivity and the cores did not enter the cytoplasm as determined by microscopy. Furthermore, cells infected with the mutant viruses were unable to form syncytia following a low pH pulse. Additional entry proteins conserved in all poxviruses were identified by mass spectroscopy of complexes captured with antibodies to the above entry proteins [71] and subsequently confirmed by constructing conditional lethal mutants. Presently, nine proteins (A16 [72], A21 [69], A28 [68,73], G3 [71,74,75], G9 [76], H2 [77,78], J5 [79], L5 [70] and O3 [67,80]) are considered to be integral components of the entry fusion complex (EFC) and two more, F9 [81] and L1 [82], have been designated EFC-associated. The phenotypes resulting from failure to express the integral or associated EFC proteins are similar and the distinction is based mainly on their contribution to the stability of the complex, which may depend on their relative locations and subunit interactions. The EFC proteins are synthesized following viral DNA replication and are dedicated to entry, since in their absence normal looking virions that are competent to synthesize mRNA *in vitro* but are unable to initiate an infection, are assembled.

The involvement of several additional VACV proteins in entry has been proposed. The phenotype of a conditional lethal I2L mutant is similar to that of EFC mutants and the I2 protein is listed in Table 1 [83]. However, the repression of I2 expression results in diminished amounts of EFC proteins in the viral membrane, raising the possibility of an indirect effect on entry [83] and association of I2 with the EFC has not been demonstrated. The heterologous expression of A17 was reported to cause fusion of transfected cells suggesting a similar role during entry [84]. A17 is a major component of the virion membrane and conditional lethal A17 mutants are blocked in viral membrane formation [85,86], where fusion may have a role, making it difficult to confirm an additional entry function.

## 6. Organization of the EFC and Structure of Subunit Proteins

The EFC has been isolated by immunoaffinity capture from non-ionic detergent-treated cytoplasmic extracts and membrane fractions of VACV-infected cells, probably representing immature virions [71]. The EFC fails to form when formation of the viral membrane is inhibited [71], thus preventing its synthesis in heterologous systems and hindering its physical characterization. The proteins are tightly bound to the membrane of the MV, making it difficult to extract as a complex even with non-ionic detergents, explaining why the infectivity of detergent extracted MVs can be partially reconstituted with lipids [47]. The EFC is destabilized when synthesis of any one of the nine integral component proteins is prevented, suggesting that it is held together by multiple subunit interactions. However, under destabilizing conditions, some subunit interactions are retained; these include interactions of A28 to H2 [78], A16 to G9 [87] and G3 to L5 [88] (Table 1). As will be detailed in a subsequent section, A16:G9 can also bind to the A56:K2 heterodimer of fusion regulatory proteins [87] and the A26 protein [119]. 

The entry proteins vary in size from 4- to 43-kDa, are non-glycosylated, and resemble neither type 1- nor type 2-fusion proteins of other viruses (Table 1). The combined mass of the EFC and EFC-associated proteins is 232 kDa, assuming each component is represented once. Five of the proteins, comprising A21, A28, G3, H2 and O3 have a N-terminal transmembrane domain; the others consisting of A16, F9, G9, J5, L1 and L5 have a C-terminal transmembrane domain. Interestingly, A16, G9, and J5 are related in sequence and apparently the progenitor was duplicated and diverged early in poxvirus evolution. Similarly, L1 and F9 are structurally related. Nevertheless each paralog is encoded by all poxviruses and is individually required for entry. With the exceptions of O3, G3 and I2, the entry proteins contain conserved intramolecular disulfide bonds that are formed by a novel cytoplasmic redox system that is encoded by all poxviruses [89]. No other viral proteins are known substrates of the poxvirus redox system, suggesting co-development with the EFC proteins perhaps because of their cytoplasmic domains. The cellular redox system, in contrast to the poxvirus system, is localized in the endoplasmic reticulum. The possibility that disulfide interchange has a role in activation of the EFC to initiate fusion is an intriguing thought, as this has been suggested for some other viruses [90,91,92,93].

Remarkably, O3 consists of only 35 amino acids, making it the smallest protein encoded by VACV [80]. The homologs in other poxviruses range from 29 to 48 amino acids in length and have a low degree of amino acid identity yet can complement an O3 deletion mutant [67]. The characteristic feature of the O3 homologs is the N-terminal transmembrane domain, which is essential and sufficient for its association with other EFC proteins [67]. 

Mutagenesis of the H2 protein defined a highly conserved region that is important for interaction with the A28 protein [78]. The A28 protein is a target of neutralizing antibodies [94] indicating an exposed location on the surface and its immunogenicity is specifically enhanced by association with H2 [95].

The EFC-associated L1 protein has been subjected to detailed analysis. L1 is a target of potent neutralizing antibodies indicating that it is exposed on the MV surface [96,97]. The protein is myristoylated at the N-terminal glycine residue [98,99] and contains three intramolecular disulfide bonds [89,100]. Mutation of the N-terminal glycine prevents the complementation of VACV infectivity [101], alters the intracellular localization of L1 as determined by confocal microscopy and reduces intramolecular disulfide bond formation [102], although the protein still associates with the EFC and MVs [103]. The crystal structure reveals a fold composed of a bundle of α-helices packed against a pair of two-stranded β-sheets [104]. The 7D11 neutralizing monoclonal antibody binds to a discontinuous epitope containing two loops that are held together by a disulfide bond [105]. Interestingly, there is a large hydrophobic cavity that could accommodate the N-terminal myristate moiety [104] and recent studies indicate that mutations within the cavity inhibit infectivity without affecting myristoylation [103]. Taken together, these results suggest a “myristate switch” model in which the acyl chain is released from the cavity during entry.

## 7. Membrane Fusion

**Figure 4:**
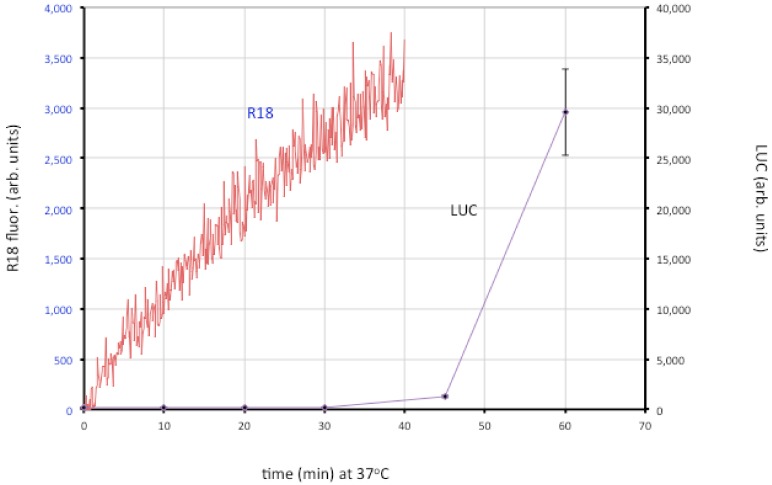
Membrane fusion and core entry. R18-loaded MVs that express firefly luciferase were incubated with HeLa cells at 4 °C to permit binding. Washed cells were then placed in a cuvette containing pre-warmed media at 37 °C and fluorescence was monitored over time (red line; left y-axis). In parallel, unlabeled MVs were bound to cells in the cold and then shifted to 37 °C. Cell lysates were prepared at indicated times and assayed for luciferase (LUC) activity (purple line; right y-axis). Modified from ref [107].

Entry of the nucleoprotein or core of enveloped viruses is usually divided into three steps: close apposition of the viral and cellular membrane, lipid-mixing of the outer membrane leaflets leading to the formation of a hemifusion intermediate, and formation and expansion of a pore [22]. The lipid-mixing step can be studied by loading the virion membrane with a self-quenching lipophilic dye such as R18 and measuring an increase in fluorescence. Fusion of VACV MVs and EVs with cells was demonstrated in that way [106]. By using recombinant VACV expressing firefly luciferase regulated by an early promoter, it has been possible to distinguish lipid-mixing and later steps dependent on core entry (Figure 4). 

The roles of individual EFC and EFC-associated proteins were investigated using MVs deficient in a single component, derived from a panel consisting of 10 conditional lethal inducible mutants [107]. The mutant virions were all able to attach to cells but most were unable to carry out even the initial lipid-mixing step. However, lipid-mixing occurred with the A28, L1 and L5 mutants but further steps did not. Two possibilities were considered: one is that the latter proteins are specifically required for pore formation or expansion; the other is that traces of A28, L1 and L5 remain in the MVs (though undetectable by Western blotting) and that this is sufficient for lipid mixing but not pore formation. Interestingly, neutralizing 7D11 monoclonal antibody to L1 also allows lipid mixing but not core entry [107]. Regardless of which hypothesis is correct, the data support a two-step model in which lipid mixing of the outer leaflets of the viral and cellular membranes occurs followed by merging of the inner leaflets and expansion of the pore to permit core entry. 

Although low pH accelerates entry of MVs as measured by the luciferase assay [25,29], lipid mixing is not enhanced by low pH [106,107]. Inhibitors of tyrosine protein kinases, dynamin GTPase and actin dynamics have little effect on binding of virions to cells but impair membrane fusion measured with R18, whereas partial cholesterol depletion and inhibitors of endosomal acidification and membrane blebbing have a more severe effect at the later stage of core entry [107]. Extensive actin remodeling and mobilization occur during MV binding to cell surfaces [37,38,108] suggesting that actin-enriched projections enhance the intimacy of membrane contact allowing virus-cell membrane fusion. Actin remodeling has been proposed to facilitate fusion by forcing membranes together and enlarging pores in a variety of systems [109,110,111] including viral protein-induced cell-cell fusion and virus entry [112,113,114,115,116,117]. In HIV entry, actin remodeling has a more important role in pore expansion and content mixing than hemifusion [118,119]. Cytochalasin D and latrunculin have a greater inhibitory effect on VACV core entry than hemifusion, suggesting that actin dynamics may be required for both hemifusion and pore formation [107].

## 8. Cell-Cell Fusion

Infection with certain VACV mutants causes cells to form syncytia. The mutations are in the A56R [120] and K2L [121,122,123] genes. Although referred to as fusion from within, syncytia formation is dependent on the formation and externalization of virions and on components of the EFC suggesting that the phenomenon has features related to virus entry [124]. A56 and K2 form a complex on the plasma membrane and the EV membrane [125]. This complex can interact with the A16 and G9 subunits of the EFC to prevent spontaneous activation of the fusion apparatus by progeny virions [87]. Uninfected cells expressing A56 and K2 but neither alone are resistant to forming syncytia when mixed with cells infected with an A56R deletion mutant [126]. Therefore, the A56-K2 complex acts as a fusion suppressor.

Wild type VACV-infected cells can also form syncytia if they are briefly exposed to low pH [106,127], similar to that occurring at neutral pH with A56R and K2L mutants. This process has also been called fusion from within, although again it is dependent on virions on the cell surface [128] and the EFC [68,69,70]. The low pH may assist in removing the outer EV membrane [129] and synchronizing the fusion process [3]. Low pH can also induce syncytium formation immediately after infection with a high multiplicity of MVs and this has been called fusion from without [127]. A recent report demonstrates that the A26 protein, like A56-K2, binds to the A16 and G9 components of the EFC, that this association is weakened by low pH, and that A26 deletion mutants can induce fusion from without at neutral pH [130]. 

## 9. Inhibition of Superinfection

VACV employs mechanisms to prevent superinfection of previously infected cells by MVs [131,132,133]. Superinfection exclusion operates between virus adsorption and early gene expression and is nearly complete by 6 h. Recent studies show that this early exclusion phenomenon occurs at the lipid-mixing step and is not dependent on expression of A56 (Laliberte, J. and Moss, B., unpublished). The association of the A16 and G9 components of the EFC with the A56-K2 complex, however, suggests that the latter represents a second mechanism of preventing superinfection as well as syncytia formation. A56 and K2 expressed by infected cells reduces the entry of superinfecting virus at late times, apparently on top of the already reduced superinfecton exclusion [134]. Moreover, uninfected cells stably expressing A56 and K2 are resistant to infection indicating that these proteins are sufficient for superinfection exclusion [126].

Superinfection exclusion also operates at the level of EVs [135]. Incorporation of VACV early proteins A33 and A36 into the plasma membrane leads to repulsion of superinfecting EVs providing a mechanism for the rapid spread of infection.

## 10. Final Thoughts

VACV has the remarkable ability to infect a wide variety of cells including those of insects, birds and mammals, although in some cases the infection is aborted following early gene expression. Perhaps the ability to infect diverse cells is a benefit of the complex poxvirus fusion apparatus and the use of alternative entry pathways. A subject that has been largely absent from this review is the existence of specific cell receptors that directly activate the viral fusion apparatus. The wide variety of permissive cells could suggest multiple or ubiquitous receptors, which would make it difficult to identify them, or alternatively the absence of such receptors. It would not be the first situation in which poxviruses demonstrate reliance on their own proteins e.g., they encode their own enzymes for gene expression, DNA replication, and disulfide bond formation. At least 11 VACV proteins are dedicated to post-attachment steps in MV entry. Although conserved in all poxviruses, these proteins have no non-poxvirus homologs nor do they resemble fusion proteins of other viruses. The combined mass of the EFC and EFC-associated proteins is 232 kDa and it is possible that several of the small proteins together form a non-linear hydrophobic face that comprises a fusion domain. A high-resolution structure of the EFC is sorely needed. The mechanism used by cell-associated EVs to enter neighboring cells is another area requiring further research. This process could be investigated using live cell imaging. Hopefully, the next review of poxvirus entry will address why so many proteins are needed rather than how many.

## Addendum

After completion of this manuscript, a related review that emphasizes poxvirus cell entry pathways was published [136].
